# Supplementary material for: Fast wide-field upconversion luminescence lifetime thermometry enabled by single-shot compressed ultrahigh-speed imaging
Source: Nat Commun. 2021 Nov 4;12:6401. doi: 10.1038/s41467-021-26701-1 (PMC8568918; doi:10.1038/s41467-021-26701-1)
Supplement: Supplementary file 1 — Supplementary Information [file 41467_2021_26701_MOESM1_ESM.pdf]

SUPPLEMENTARY MATERIALS

Fast wide-field upconversion luminescence lifetime thermometry enabled by single-shot compressed ultrahigh-speed imaging

Xianglei Liu[1,†], Artiom Skripka[1,2,†], Yingming Lai[1], Cheng Jiang[1], Jingdan Liu[1], Fiorenzo Vetrone[1,*], Jinyang Liang[1,*]

[1]Centre Énergie Matériaux Télécommunications, Institut National de la Recherche Scientifique, 1650 boulevard Lionel-Boulet, Varennes, Québec J3X1S2, CANADA

[2]Present address: Nanomaterials for Bioimaging Group, Departamento de Física de Materiales, Facultad de Ciencias, Universidad Autónoma de Madrid, Madrid, 28049, SPAIN and The Molecular Foundry, Lawrence Berkeley National Laboratory, Berkeley, California 94720, USA

[†]These authors contributed equally to this work

*Corresponding authors: Fiorenzo.Vetrone@inrs.ca (F.V.) and Jinyang.Liang@inrs.ca (J.Liang)

Supplementary Note 1: Two-view image registration of the SPLIT system

To conduct the image registration between the two views, we used an established procedure [1] to calibrate the single-shot photoluminescence lifetime imaging thermometry (SPLIT) system. In particular, a static target of upconverting nanoparticles (UCNPs) was imaged by the SPLIT system to form View 1 and View 2. No optical shearing was performed in the recording of View 2. The projective transformation was then quantified by using the registration estimator toolbox in MATLAB R2019b [2], which supplied a feature-based registration operator to automatically detect distinct local features, such as sharp corners, blobs, or regions of images. The transformation matrix $\mathbf{P}_{\mathrm{tm}}$ is defined as

$$\mathbf{P}_{\mathrm{tm}} = \begin{bmatrix} s_x \cos\theta & -s_y \sin\theta & l_x \\ s_x \sin\theta & s_y \cos\theta & l_y \\ 0 & 0 & 1 \end{bmatrix}. \quad (1)$$

Here s_x and s_y are the scaling factors in the x direction and the y direction. θ is the rotation angle. l_x and l_y represent translation factors in the x direction and the y direction, respectively. Each pixel in View 1 with a homogeneous coordinate $[h_x\ h_y\ 1]$ is transformed to the corresponding point $[h_{xc}\ h_{yc}\ 1]$ by

$$\left[h_{xc}\ h_{yc}\ 1\right]^T = \mathbf{P}_{\mathrm{tm}}\left[h_x\ h_y\ 1\right]^T. \tag{2}$$

In practice, $\mathbf{P}_{\mathrm{tm}}$ was computed by using the static letter "A" pattern. Supplementary Figs. 1a–b show the acquired images in View 1 and View 2. The co-registered View 1 image (Supplementary Fig. 1c) and the View 2 image were used for SPLIT's image reconstruction.

Supplementary Note 2: Derivation of the SPLIT's reconstruction algorithm

In image reconstruction, the datacube of the dynamic scene is recovered by solving the minimization problem aided by regularizers [3]. In particular, the inverse problem [i.e., Equation (2) in Main Text] is first written as

$$\begin{gathered}\hat{I} = \underset{I,v,u,w\in\mathbf{A}}{\operatorname{argmin}}\left\{\frac{1}{2}\|\mathbf{T}v - E\|_2^2 + R(u) + \mathbf{I}_+(w)\right\} \\ \text{subject to } v = \mathbf{M}I, u = I, w = I,\end{gathered} \tag{3}$$

where v, u, and w are primal variables. **A** is the set of possible solutions in compliance with the spatial constraint [4], which is generated by binarizing the image of View 1 (i.e., E_1) with an appropriate intensity threshold that is determined by the Otsu's method [5]. Then, Supplementary Equation (3) is further written in the augmented Lagrangian form [6-8]:

$$\begin{gathered}\hat{I} = \underset{I,v,u,w\in\mathbf{A}}{\operatorname{argmin}}\left\{\frac{1}{2}\|\mathbf{T}v - E\|_2^2 + R(u) + \mathbf{I}_+(w)\right. \\ \left. + \frac{\mu_1}{2}\|\mathbf{M}I - v + \frac{\gamma_1}{\mu_1}\|_2^2 + \frac{\mu_2}{2}\|I - u + \frac{\gamma_2}{\mu_2}\|_2^2 + \frac{\mu_3}{2}\|I - w + \frac{\gamma_3}{\mu_3}\|_2^2\right\}.\end{gathered} \tag{4}$$

Here, γ_1, γ_2, and γ_3 are dual variables. μ_1, μ_2, and μ_3 are penalty parameters [9,10]. The block-matching and 3D (BM3D) filtering [11] is used as the plug-and-play (PnP) denoiser in the implicit regularizer $R(\cdot)$. The ramp function [12] is used in the non-negative indicator function $\mathbf{I}_+(\cdot)$.

To retrieve the dynamic scene, the reconstruction algorithm sequentially updates primal variables, estimated solution I^{k+1} (k denotes the iteration time), dual variables and penalty parameters as well as evaluates the pre-set criteria, as following five steps.

Step 1: update primal variables (i.e., v, u, and w) by

$$v^{k+1} = (\mathbf{T}^T \cdot \mathbf{T} + \mu_1^k \mathrm{D})^{-1} \cdot \left(\mathbf{T}^{\mathrm{T}} E + \mu_1^k \mathbf{M} I^k + {\gamma_1}^k\right),$$
$$u^{k+1} = \mathrm{D}_{\mathrm{BM3D}}(I^k + \frac{{\gamma_2}^k}{\mu_2^k}), \quad \text{and} \tag{5}$$
$$w^{k+1} = \max\{0, I^k + \frac{{\gamma_3}^k}{\mu_3^k}\}.$$

Here, D is the identity matrix. $\mathrm{D}_{\mathrm{BM3D}}(\cdot)$ stands for the BM3D filtering [11].

Step 2: update the estimated datacube of the dynamic scene [i.e., $I(x, y, t)$] by

$$I^{k+1} = \left(\mu_1^k \mathbf{M}^T \cdot \mathbf{M} \cdot \mathrm{D} + \mu_2^k \mathrm{D} + \mu_3^k \mathrm{D}\right)^{-1} \left[\mu_1^k \mathbf{M}^T (v^{k+1} - \frac{{\gamma_1}^k}{\mu_1^k}) + \mu_2^k (u^{k+1} - \frac{{\gamma_2}^k}{\mu_2^k}) + \mu_3^k (w^{k+1} - \frac{{\gamma_3}^k}{\mu_3^k})\right]. \tag{6}$$

Step 3: update dual variables (i.e., γ_1, γ_2, and γ_3) by

$${\gamma_1}^{k+1} = {\gamma_1}^k + \mu_1^k (\mathbf{M} I^{k+1} - v^{k+1}),$$
$${\gamma_2}^{k+1} = {\gamma_2}^k + \mu_2^k (I^{k+1} - u^{k+1}), \quad \text{and} \tag{7}$$
$${\gamma_3}^{k+1} = {\gamma_3}^k + \mu_3^k (I^{k+1} - w^{k+1}).$$

Step 4: update the penalty parameters (i.e., μ_1, μ_2, and μ_3) by

$$\mu_i^{k+1} = \begin{cases} \varphi \mu_i^k, & \text{if } p > \sigma q \\ \dfrac{\mu_i^k}{\varphi}, & \text{if } \sigma p < q \\ \mu_i^k, & \text{otherwise} \end{cases} \quad (i = 1, 2, 3). \tag{8}$$

Here, $p = \|I^{k+1} - v^{k+1}\|_2$ is the primal residual, and $q = \mu_i^k \|I^{k+1} - I^k\|_2$ is the dual residual. φ ($\varphi > 1$) is the balancing factor, and σ ($\sigma > 1$) is the residual tolerance [13]. In our experiments, we chose $\varphi = 1.1$ and $\sigma = 1.5$.

Step 5: judge the relative change in results and the parameters μ_1^{k+1}, μ_2^{k+1}, and μ_3^{k+1} in adjacent iterations by

$$\text{if } \eta = \frac{\|I^{k+1} - I^k\|_2}{\|I^{k+1}\|_2} < \rho \text{ and } \mu_i^{k+1} = \mu_i^k \ (i = 1, 2, 3). \tag{9}$$

Here, ρ ($0 < \rho < 10^{-3}$) is the pre-set tolerance value. These steps are repeated until both criteria in Step 5 are satisfied. The image reconstruction recovers the datacube of the dynamic scene.

Supplementary Note 3: Simulation results of the dual-view PnP- ADMM algorithm

To test the proposed dual-view plug-and-play alternating direction method of multipliers (PnP-ADMM) algorithm, we reconstructed a simulated dynamic scene—the intensity decay of a static Shepp-Logan phantom. This dynamic scene contained 12 frames, each with a size of 200×200 pixels. The intensity in each frame was determined by a single exponential function of $I_{n_t} = \exp[-(n_t - 1)/2]$, where $n_t = 1, \dots, 12$ denotes the frame index.

Then, this dynamic scene was fed into SPLIT's forward model [i.e., Equation (1) in Main Text] to generate E_1 and E_2. To mimic the experimental conditions, we added Gaussian noise (0.01 variance and 0 mean value) into E_1 and E_2. Finally, these two images were input into the dual-view PnP-ADMM algorithm to retrieve the datacube of this dynamic scene. Representative reconstructed frames and their corresponding ground truth frames are compared in Supplementary Fig. 2a. The averaged peak signal-to-noise ratio and the averaged structural similarity index over all reconstructed images were calculated to be 34.6 dB and 0.96, respectively. The reconstructed three local features in Frame 1 are compared to their ground truths (Supplementary Fig. 2b). Supplementary Fig. 2c presents the reconstructed normalized average intensity versus time, which has a good agreement with the pre-set intensity decay (black dashed line).

Supplementary Note 4: Details on the relationship between temperature and lifetime

The normalized area integration method is commonly used for calculating lifetime based on pulsed excitation [14]. Photoluminescence lifetime of UCNPs following pulsed excitation can be expressed by

$$\mathrm{L_t} = \int_0^\infty f(t) * g(t) dt. \tag{10}$$

Here $f(t) = \frac{1}{\sqrt{\pi} t_\mathrm{w}} \exp\left(-\frac{t^2}{t_\mathrm{w}^2}\right)$ represents the Gaussian excitation pulse with a pulse width of t_w. $g(t) = \sum \varepsilon_i \exp\left(-t/\tau_i\right)$ is used to represent the photoluminescence with multiple exponential decays, each of which has a lifetime τ_i and a proportion ε_i. "$*$" denotes convolution. Then, Supplementary Equation (10) becomes

$$\mathrm{L_t} = \sum \varepsilon_i \tau_i \exp\left(\frac{t_\mathrm{w}^2}{4\tau_i^2}\right). \tag{11}$$

When t_w approaches to zero, which indicates the case of an ultrashort pulse, Supplementary Equation (11) becomes

$$\mathrm{L_t} = \sum \varepsilon_i \tau_i. \tag{12}$$

Following the established theory [15], we defined the photoluminescence lifetime as $\tau = \sum \varepsilon_i \tau_i / \sum \varepsilon_i$. Considering that $\sum \varepsilon_i = 1$, we have $\tau = \mathrm{L_t}$.

The lifetime is linearly linked to the temperature by

$$T = c_\mathrm{t} + \frac{\mathrm{L_t}}{S_\mathrm{a}}. \tag{13}$$

Here S_a denotes the absolute temperature sensitivity, and c_t denotes a constant. This derivation produces Equation (3) in Main Text.

In the SPLIT system, we used a continuous-wave laser and an optical chopper to generate excitation pulses. Although the chopper blade's slit width could approach zero for generating an ultrashort pulse duration, it demands a high laser power. Thus, a finite pulse width needs to be chosen to provide sufficient signal-to-noise ratio (SNRs) in measurements while still maintaining accurate lifetime calculation. In practice, we chose $t_\mathrm{w} = 50\ \mu\mathrm{s}$, which was comparable to the values used in the literature [14]. Our calculation also showed that this pulse width induced a <0.3% calculation error for the 5.6-mm-thick-shell UCNPs that were mainly used in our experiments. Thus, 50-μs pulse width allowed SPLIT to produce accurate temperature mapping results.

Supplementary Note 5: Preparation and characterization of UCNPs

Synthesis of UCNPs

Core/shell $NaGdF_4$: 2 mol% Er^{3+}, 20 mol% Yb^{3+}/$NaGdF_4$ UCNPs were synthesized via the previously reported thermal decomposition method, with minor modifications to the synthesis procedure [16]. Core precursors were prepared by mixing 0.025 mmol of Er_2O_3 (REacton 99.99%), 0.250 mmol Yb_2O_3 (REacton 99.99+%), and 0.975 mmol Gd_2O_3 (REacton 99.99+%) with 5 mL trifluoroacetic acid (99%) and 5 mL of distilled water in a 50 mL three-neck round bottom flask. Shell precursors were prepared separately by mixing 1.5 mmol of Gd_2O_3 with 5 mL of trifluoroacetic acid and 5 mL of distilled water in a 50 mL three-neck round bottom flask. Mixtures were refluxed under vigorous stirring at 80 °C until each solution turned from turbid to clear, at which point the temperature was decreased to 60 °C to slowly evaporate the excess trifluoroacetic

acid and water. All precursors were obtained as solid dried materials and were used for the UCNPs synthesis without further purification. All materials involved in the precursor synthesis (obtained from Alfa Aesar) were used without further purification.

The first step was to synthesize the core UCNPs. An initial mixture of 12.5 mL each of oleic acid (OA; 90%, Alfa Aesar) and 1-octadecene (ODE; 90%, Alfa Aesar) was prepared in a 100 mL three-neck round bottom flask (Solution A). Aside, 2.5 mmol of sodium trifluoroacetate (98%, Alfa Aesar) was added to the dried core precursor together with 7.5 mL each of oleic acid and 1-octadecene (Solution B). Both Solutions A and B were degassed at 145 ºC under vacuum with magnetic stirring for 30 minutes. After degassing, Solution A was placed under an inert Ar atmosphere and the temperature was slowly raised to 315 ºC. Solution B was then injected into the reaction vessel containing Solution A using a syringe and pump system (Harvard Apparatus, Pump 11 Elite) at a 1.5 mL min^{-1} injection rate. The mixture was left at 315 ºC under vigorous stirring for 60 minutes. The synthesized core UCNPs were stored in Falcon centrifuge tubes (50 mL) under Ar for the further shelling step. Due to the evaporation of impurities in starting materials (e.g., OA and ODE) and reaction byproducts, as well as minor losses accrued from intermediate steps of liquid handling, the final volume of the core mixture was around 36 mL.

In the second step, core/shell UCNPs of different shell thicknesses were prepared by epitaxial growth of the shell on the preformed cores via a multi-step hot-injection approach. First, we prepared Solution A by mixing approximately 1.5 mmol of core UCNPs (~21.6 mL) in a 100 mL three-neck round bottom flask together with 9.2 mL each of OA and ODE. Separately, we prepared Solution B by mixing 3 mmol of gadolinium trifluoroacetate (shelling) precursors with 3 mmol of sodium trifluoroacetate, and 10.5 mL each of OA and ODE. Both solutions were degassed under vacuum and magnetic stirring at 110 ºC for 30 minutes. After degassing, Solution A was back-filled with argon gas and the temperature was raised to 315 ºC. Solution B was then injected into the reaction vessel containing Solution A using a syringe and pump system at a 0.75 mL min^{-1} injection rate in three steps. After each ~7 mL injection step, the mixture was allowed to react for 60 minutes. A portion of core/shell UCNPs would be extracted before the next injection step: 15.6 mL after the first injection step for core/shell UCNPs with a 1.9 nm-thick shell and 19.2 mL after the second injection step for core/shell UCNPs with a 3.5 nm-thick shell. Extractions were allowed to cool down to room temperature before transfer from glass syringe to Falcon centrifuge tube for subsequent washing. After the final injection step and a total of 180

minutes of reaction, the mixture (core/shell UCNPs with a 5.6 nm-thick shell) was cooled to room temperature under argon gas and magnetic stirring. All core/shell UCNPs were precipitated with ethanol and washed three times with hexane/acetone (1/4 v/v in each case), followed by centrifugation (with 5400 x g). Finally, all UCNPs were re-dispersed in hexane for further structural and optical characterization.

Structural characterization

The morphology and size distribution of the core/shell UCNPs were investigated by transmission electron microscopy (TEM, Philips, Tecnai 12). The particle size was determined from TEM images using ImageJ software with a minimum set size of 280 individual UCNPs per sample. The results are shown in Figure 2a. The crystallinity and phase of the core-only and core/shell UCNPs were determined via X-ray powder diffraction (XRD) analysis using a diffractometer (Bruker, D8 Advance) with CuKα radiation (Supplementary Fig. 3). The peaks in measured XRD spectra match the reference tabulated data (PDF# 01-080-8787). Along with the TEM images (i.e., Figure 2a), this result ensured that the fabricated UCNPs were of the hexagonal crystal phase.

Supplementary Note 6: Characterization of SPLIT's system sensitivity

To test the sensitivity of SPLIT, we monitored the reconstructed image quality while decreasing the laser power. The detection sensitivity of the SPLIT system was characterized by imaging photoluminescence intensity decay with various excitation power densities (Supplementary Fig. 4). Transparency of the letter "P" covered the sample of UCNPs with a shell thickness of 5.6 nm. The laser power density was varied from 0.4 to 0.04 W mm^{-2}. All other experimental parameters, such as exposure time, camera gain, and temperature, were kept the same. The quality of reconstructed images kept degrading with decreased laser power density until partially losing spatial structure at <0.06 W mm^{-2}. In addition, lower SNRs in measurements deteriorate the image reconstruction, manifested by the increase in noise levels in the intensity decay curves and the deviation of the calculated photoluminescence lifetime from the correct values. Thus, the SPLIT's sensitivity under single-shot imaging for this UCNP sample was quantified to be 0.06 W mm^{-2}.

Supplementary Note 7: Measurement of photoluminescence lifetimes of UCNPs using the time-correlated single-photon counting (TCSPC) technique

To ascertain our results, we used the standard TCSPC method (Edinburgh Instruments, FLS980, 70-µs excitation pulse) to measure photoluminescence decay of the 5.6 nm-thick-shell UCNPs dispersed in hexane. The measured intensity decay curve is shown in Supplementary Fig. 5. Lifetime values acquired from the SPLIT and TCSPC measurements yielded a 6.9% mismatch. This difference is attributed to different environments in which UCNPs were measured (dried powder for SPLIT and solution for TCSPC), different excitation pulse widths (50-µs for SPLIT and 70-µs for TCSPC), and different instrumental responses.

Supplementary Note 8: Comparison of reconstructed image quality

To quantitatively demonstrate the superiority of the dual-view PnP-ADMM algorithm employed in SPLIT's image reconstruction, we compared it with two other algorithms dominantly used in existing streak-camera-based single-shot ultrafast imaging—the single-view two-step iterative thresholding/shrinkage (TwIST) algorithm [21] and the dual-view TwIST algorithm [22-24]. Specifically, we used the experimental data of the green emission of UCNPs with shell thicknesses of 1.9 nm, 3.5 nm, and 5.6 nm, covered by transparencies of letters "C", "A", and "N", respectively. Both View 1 and View 2 were used for the dual-view TwIST algorithm and the dual-view PnP-ADMM algorithm. Only View 2 was used for the single-view TwIST algorithm. All the reconstructed datacubes had the same size. Supplementary Figs. 6a–c show the time-integrated images by projecting datacubes reconstructed by the three algorithms along the time axis. Among them, the result from the dual-view PnP-ADMM is duplicated from Figure 2f to better illustrate this comparison. We selected one line from each letter and compared their profiles in Supplementary Figs. 6d–f. From these results, the single-view TwIST algorithm gives the worst contrasts of 0.41 for "C", 0.88 for "A", and 0.76 for "N". Dual-view TwIST improves the contrast to 0.82 for "C", 0.99 for "A", and 0.93 for "N", respectively. Dual-view PnP-ADMM gives the best result—producing contrasts of 1 for all three cases.

The better quality in the reconstructed images translated to higher accuracy in lifetime quantification. Supplementary Figs. 6g–i show two-dimensional (2D) lifetime maps of these samples with zoom-in-views of three local areas. Both the single-view and dual-view TwIST

algorithms yield artifacts, manifesting as false lifetime values on pixels in the background. In contrast, the dual-view PnP-ADMM algorithm eliminates these artifacts with a clean background. Meanwhile, in the selected local areas of letters "C" and "N" (insets in Supplementary Figs. 6g–i), single-view TwIST completely wipes out the features induced by the non-uniform distribution of the UCNPs. In contrast, both dual-view TwIST and dual-view PnP-ADMM algorithms preserve these features. Finally, benefitting from the superb denoising capability of the dual-view PnP-ADMM, the noise level in the intensity decay curves as a function of time reduces by 4.6× and 2.5× compared to those of the single-view TwIST and dual-view TwIST algorithms, which contributes to a more accurate lifetime calculation.

Supplementary Note 9: Determination of the absolute temperature sensitivities, relative temperature sensitivities, and thermal uncertainty

Both the absolute temperature sensitivity S_a and the constant c_t in Equation (3) in Main Text are determined by using the curve fitting toolbox in Matlab. Using the data presented in Figure 3e with linear fitting, we quantified $S_\mathrm{a} = -1.90\ \mu\mathrm{s}\ {}^\circ\mathrm{C}^{-1}$ and $c_\mathrm{t} = 278\ {}^\circ\mathrm{C}$ for the green emission and $S_\mathrm{a} = -2.40\ \mu\mathrm{s}\ {}^\circ\mathrm{C}^{-1}$ and $c_\mathrm{t} = 210\ {}^\circ\mathrm{C}$ for the red emission.

Moreover, the relative temperature sensitivity can be calculated by [17]

$$S_\mathrm{r} = \frac{|S_\mathrm{a}|}{\tau}. \tag{14}$$

Using the data shown in Figure 3e, S_r in the pre-set temperature range were quantified to be 0.39–0.43%·$^\circ$C^{-1} for the green emission and 0.52–0.60%·$^\circ$C^{-1} for the red emission (Supplementary Fig. 7).

Finally, the thermal uncertainty [17] in SPLIT is calculated by

$$\delta T = \frac{1}{S_\mathrm{r}} \times \frac{\delta\tau}{\tau}, \tag{15}$$

where $\delta\tau$ represents the uncertainty in the measured lifetimes. Supplementary Equation (15) shows that δT depends on both the UCNPs' performance (quantified by the relative sensitivity, S_r) and experimental setup (that limits the normalized fluctuation of lifetimes, $\frac{\delta\tau}{\tau}$). $\delta\tau$ was characterized by repeating measurements using the SPLIT system under the same experimental conditions. Specifically, using the sample of the 5.6 nm shell thickness UCNPs at 20 $^\circ$C, we

repeated the 2D lifetime measurements 60 times using the excitation power density of 0.4 W mm^{-2} and 0.06 W mm^{-2}, respectively. These measurements produced $\delta\tau$ of 1.4–2.7 μs for the green emission and 2.2–4.0 μs for the red emission, respectively. With known values of $|S_{\mathrm{a}}|$ and by using Supplementary Equation (15), SPLIT's thermal uncertainty was calculated to be 0.7–1.4 ºC for the green emission and 0.9–1.7 ºC for the red emission.

Supplementary Note 10: Demonstration of SPLIT in biological environment

The UCNP sample with the shell thickness of 5.6 nm was covered by lift-out grids (Ted Pella, 460-2031-S), in which we chose the features of the letter "O" with a triangular shape on the bottom and the letter "m". Then, five pieces of fresh chicken tissue with the thicknesses of 0.25, 0.5, 0.65, 0.75, 1.0 mm were used to cover the sample (Supplementary Fig. 8a). SPLIT captured the photoluminescence decay at 20 kfps. The reconstructed datacubes were projected to the $x - y$ plane (Supplementary Figs. 8b-c). The full evolution of intensity decay of the green and red emissions covered by lift-out grids beneath chicken tissue with different thicknesses is in Supplementary Movie 4.

The image without chicken tissue, which is referred to as the thickness of "0 mm", is also included for comparison. With the increased depth, the image intensity and contrast gradually approach zero. Supplementary Fig. 8d depicts the normalized fluence profiles across the white dashed line as shown in the first panel of Supplementary Fig. 8b. The experimental result was fitted using a single-component exponential function, which yielded a decay coefficient of 26 cm^{-1}. At the depth of 0.65 mm, the triangular feature and the letter "m" cannot be distinguished. Using a similar experimental procedure, we characterized SPLIT's imaging depth for the red emission (Supplementary Figs. 8c and 8e). By using the single-component exponential fitting, the red emission had a decay coefficient of 18 cm^{-1}. The spatial features vanished at the depth of 0.75 mm. These results show that the red emission has, as expected, a greater imaging depth than the green upconversion counterpart. These results also show that good contrast can be maintained by using fresh chicken tissue of 0.5 mm thickness, which was selected for the longitudinal temperature monitoring experiments (Supplementary Fig. 9).

To test SPLIT using a scattering medium with the presence of both light scattering and absorption, the UCNPs with the shell thickness of 5.6 nm were injected into a piece of fresh beef

tissue, where we also inserted a 90 µm-diameter copper wire at the depth of 0.09 mm as a spatial feature (Supplementary Fig. 10a). Myoglobin in the beef tissue, which has similar optical absorption properties to hemoglobin [18,19], was used to mimic the absorption by blood. To evaluate the SPLIT's imaging ability at different depths, this phantom was covered by different additional fresh beef slices, so that the thicknesses from the surface to the copper wire were 0.09 mm, 0.34 mm, 0.55 mm, and 0.60 mm. SPLIT performed photoluminescence lifetime imaging at 20 kfps. For both the green and the red emissions, the reconstructed datacubes with the different beef thicknesses were projected temporally, as shown in Supplementary Figs. 10b–c. Furthermore, we plotted the profiles of a selected local edge feature of the inserted copper wire under the different thicknesses, as shown in Supplementary Figs. 10d–e. We calculated the contrast of these edge profiles. For the green emission, the values are 0.78, 0.27, 0.26, and 0.09 for the four selected curves. As for the red emission, these values are 0.80, 0.38, 0.33, and 0.09. Moreover, these experimental results were fitted by using single-component exponential functions, which yielded decay coefficients of 65 cm^{-1} for the green emission and 33 cm^{-1} for the red emission (Supplementary Fig. 10f), which are greater than their counterpart of the chicken tissue of 26 cm^{-1} and 18 cm^{-1}. Because of its longer wavelength, the red emission has weaker scattering and weaker absorption by the myoglobin, which led to deeper penetration over the green emission for both types of scattering media. Finally, we analyzed the photoluminescence lifetimes for different thicknesses, and the results are shown in Supplementary Fig. 10g. The measured photoluminescence lifetimes for both emissions do not depend on the tissue thickness and hence excitation power density under the experimental conditions of our work. Lower excitation intensity, however, reduced the SNRs in the captured snapshots, which transfers to a larger standard deviation.

Supplementary Note 11: Preparation of the single-layer onion cells doped with UCNPs

For the onion cell experiments, UCNPs with a 5.6 nm-thick shell were first transferred to water via ligand exchange with citrate molecules. In a typical procedure, citrate-coated UCNPs were prepared by mixing 50 mg of oleate-capped UCNPs dispersed in 25 mL of hexane and 25 mL of 0.2 M trisodium citrate (99%; Alfa Aesar) solution (pH 3-4) under vigorous stirring for 3 hours. The two-phase (aqueous/organic) mixture was then poured into the separatory funnel, and the

aqueous phase containing the UCNPs was isolated. The UCNPs were precipitated with acetone (1/3 v/v) via centrifugation (5400 x g) for 30 minutes. The obtained pellet was re-dispersed in 25 mL of 0.2 M trisodium citrate solution (pH 7-8) and left under stirring for an additional 2 hours. UCNPs were then precipitated with acetone (1/3 v/v) via centrifugation (5400 x g) for 30 minutes and washed twice with a mixture of water/acetone (1/3 v/v). The citrate-coated UCNPs were re-dispersed in distilled water. The yellow household onion was used to peel single-layer sheets of onion cells, which were incubated in a solution of citrate-coated UCNPs (3 mg mL^{-1}) for 24 hours. After the incubation, single-layer onion cells were rinsed in distilled water and dried by gently tapping with a soft tissue paper, before being placed onto microscope slides for subsequent imaging experiments. Before lifetime imaging, the presence of UCNPs in single-layer onion cells was confirmed (Supplementary Fig. 11a) with a bright-field microscope (Nikon, ECLIPSE Ti-S). In addition, a reference photoluminescence intensity image was taken by a custom-built confocal imaging platform (Photon Etc.), equipped with pulsed femtosecond Ti: Sapphire laser (Spectra-Physics, Mai Tai DeepSee). Samples were excited and imaged epi-fluorescently through a 20×/0.40 NA objective lens (Nikon, CFI60 TU Plan Epi ELWD). Photoluminescence intensity was recorded by a low-noise CCD camera (Princeton Instruments, Pixis100). The upconversion emission images of static onion cells (Supplementary Fig. 11b) were obtained through raster scanning a 120×120 pixel map, each of which has the size of 2 μm and the integration time 0.2 seconds per pixel. The total time to form one lifetime map was 48 minutes.

Supplementary Note 12: Comparison between SPLIT and previous streak-camera-based modalities for 2D lifetime imaging

To articulate the difference between SPLIT and previous works on ultrafast imaging that used streak cameras, we summarize their technical specifications and applications in Supplementary Table 1. To explain the details included in this table, we first detail the working principles of streak cameras and compressed ultrafast photography (CUP); then, we summarize technical specifications and applications of the existing imaging modalities.

Streak cameras for wide-field lifetime imaging

Streak cameras are highly suitable for 2D lifetime imaging. In its conventional operation, the field of view (FOV) of streak cameras is limited by an entrance slit with typical widths of 50–100 µm. A sweeping unit deflects the time-of-arrival of the incident light signal along the axis perpendicular to the device's entrance slit. Depending on the mechanisms of the sweeping unit, streak cameras can be generally categorized into optoelectronic and mechanical types. In optoelectronic streak cameras (Supplementary Fig. 12a), incident photons are first converted to photoelectrons by a photocathode. After acceleration, these photoelectrons are deflected by a time-varying voltage applied on a pair of sweep electrodes. Then, these photoelectrons are converted back to photons on a phosphor screen. Finally, the optical signal is imaged to an internal sensor. The optoelectronic streak camera can achieve a temporal resolution of up to 100 fs. Because of this ultrafast imaging ability, optoelectronic streak cameras have been used for imaging the emission of fluorescence that has lifetimes in the order of picoseconds and nanoseconds [20-24]. However, due to the photon-to-photoelectron conversion by the photocathode, the quantum efficiency (QE) of the optoelectronic streak cameras is typically <15% for visible light. Besides, the space-charge effect in the electrostatic lens system imposes constraints in the spatial resolution (typically tens to hundreds of micrometers) and the dynamic range (e.g., <10 for certain femtosecond streak cameras). Both weaknesses severely limit the quality of acquired data.

Unlike optoelectronic streak cameras, a mechanical streak camera (Supplementary Fig. 12b) usually uses a rotating mirror (e.g., a galvanometer scanner or a polygon mirror) to deflect the light. Since the mechanical sweeping is much slower than the optoelectronic counterpart, this type of streak camera has tunable temporal resolutions typically from hundreds of nanoseconds to microseconds, which makes them highly suitable for lifetime imaging of luminescence processes on the order of microseconds and milliseconds, such as phosphorescence and parity forbidden 4f-4f transitions in lanthanide ions [25]. Moreover, its all-optical data acquisition allows flexibly implementing many high-sensitivity cameras [e.g., electron-multiplying (EM) CCD and scientific CMOS cameras, whose QEs can be >90% for visible light] to obtain superior SNRs in measurements. The all-optical operation also avoids the space-charge effect, which enables optics-limited spatial resolution and high dynamic range (e.g., >60,000 of the EMCCD camera used in this work). Finally, the mechanical streak camera is considerably more cost-efficient than the

optoelectronic streak camera. Therefore, mechanical streak cameras are perfectly suitable for imaging microsecond-level emission from UCNPs.

Single-shot compressed temporal imaging for fast 2D lifetime mapping

Single-shot compressed temporal imaging is a novel computational imaging concept that enables 2D lifetime mapping in one acquisition. In the conventional operation of the streak camera, the entrance slit limits the imaging FOV to be one-dimensional (1D). To lift this limitation, compressed-sensing paradigms have been implemented with optoelectronic streak cameras. The resulted CUP technique [21-24] allows complete opening of the entrance slit for 2D ultrafast imaging in a single shot. CUP and its variants have been applied to single-shot fluorescence lifetime imaging [21,23,24]. In contrast, to our knowledge, single-shot compressed temporal imaging has not yet been applied to 2D imaging of microsecond-to-millisecond scale lifetimes, like those of UCNP emission. SPLIT thus marks the first technique in this category. It is also the first demonstration of single-shot photoluminescence lifetime-based temperature mapping in a 2D FOV. Compared to conventional line-scanning counterpart [25], SPLIT has considerable advantages in light throughput and sample choices.

Performance comparison between SPLIT and line-scanning lifetime imaging

To experimentally demonstrate the advantages of SPLIT to the line-scanning confocal setup [25], we imaged a moving photoluminescent sample (Supplementary Fig. 13). The major experimental parameters (e.g., magnification ratio, camera's exposure time, and camera's frame rate) were kept the same as those of SPLIT. The UCNPs with the shell thickness of 5.6 nm were covered by a piece of transparency of letter "A". Loaded onto a translation stage, this sample moved downward at a speed of 0.8 mm s^{-1}. To perform line scanning, we placed a 200-µm-wide slit at the intermediate image plane (i.e., equivalently 50-µm-wide at the sample plane) to limit the FOV to 1D (Supplementary Fig. 13a). Attached to another translation stage, the slit was scanned in the x direction at a speed of 2.8 mm s^{-1}. Using the green emission, this line-scanning confocal setup generated six 1D lifetime maps (Supplementary Fig. 13b). After stitching these results together, we obtained a 2D lifetime map as shown in Supplementary Fig. 13c. However, the stitched result inevitably suffers from the loss of spatial content due to the dark time between adjacent camera exposures. In the meantime, the map is distorted in the vertical direction due to the sample's

movement, which proves the incapability of line-scanning-based techniques in measuring dynamic photoluminescent objects. As a comparison, we used SPLIT to image this sample under the same experimental conditions. Because of its single-shot imaging ability, SPLIT produced six 2D lifetime maps (Supplementary Fig. 13d). No image produced by the SPLIT system has any loss of spatial content or distortion. The results also clearly illustrate the downward movement of the letter "A". Therefore, SPLIT has unique advantages over the conventional scanning-based lifetime measurement in data throughput, measurement accuracy, and application scope.

It is also worth pointing out that from the perspective of optical instrumentation, SPLIT provides high-sensitivity cameras with ultrahigh imaging speeds in 2D FOV. In this regard, besides the single-shot wide-field photoluminescent lifetime mapping demonstrated in this work, the SPLIT system offers a generic imaging platform for many other studies. Potential future applications include optical voltage imaging of action potentials in neurons and high-throughput flow cytometry.

Supplementary Note 13: Comparison between SPLIT and thermal imaging

We used a thermal imaging camera (Yoseen, X384D) (Supplementary Fig. 14a) and SPLIT (Supplementary Fig. 14b) to image UCNPs covered by a metal mask of letters "rob" in lift-out grids (Ted Pella, 460-2031-S). Akin to the SPLIT system, a 4× magnification ratio was used for the thermal imaging camera. A blackbody radiator (Yoseen, YSHT-35) was used to heat this sample to 27 ºC. The images produced by these two methods are shown in Supplementary Figs. 14c–d and the selected line profiles are shown in Supplementary Figs. 14e–f. The edge contrast of the imaged letters using the thermal imaging camera is much worse than that using SPLIT. Moreover, the thermal imaging result presents strong background due to the same temperature of the mask, whereas SPLIT keeps a clean background thanks to its optical sensing ability.

In another experiment, we loaded the metal mask on a translation stage. The mask was kept out of the FOV to keep its temperature at 18 °C (i.e., the room temperature in our laboratory). The UCNPs were still heated up by the blackbody radiator to 27 °C. The mask was quickly moved into the FOV, and the thermal imaging camera captured the images immediately (Supplementary Fig. 14g). The thermal image and the selected line profiles are shown in Supplementary Figs. 14h–i. Despite the slight improvement in contrast compared to Supplementary Figs. 14c and 14e, the

image quality is still incomparable to the results produced by the SPLIT system (Supplementary Figs. 14d and 14f). Thus, compared to a thermal imaging camera, SPLIT supplies superior temperature mapping capability.

Supplementary References

1. Jiang C, Kilcullen P, Lai Y, Ozaki T, Liang J. High-speed dual-view band-limited illumination profilometry using temporally interlaced acquisition. *Photonics Res.* **8**, 1808-1817 (2020).
2. The MathWorks Inc. Register Images Using Registration Estimator App. (2020). URL: https://www.mathworks.com/help/images/register-images-using-the-registration-estimator-app.html. Accessed on 2021-07-04.
3. Antipa N, *et al*. DiffuserCam: lensless single-exposure 3D imaging. *Optica* **5**, 1-9 (2018).
4. Zhu L, *et al*. Space- and intensity-constrained reconstruction for compressed ultrafast photography. *Optica* **3**, 694-697 (2016).
5. Otsu N. A threshold selection method from gray-level histograms. *IEEE Trans. Syst. Man Cybern. Syst.* **9**, 62-66 (1979).
6. Lai Y, *et al.* Single-Shot Ultraviolet Compressed Ultrafast Photography. *Laser Photonics Rev.* **14**, 2000122 (2020).
7. Nocedal J, Wright S. Sequential quadratic programming. *Numerical Optimization*, 529-562 (Springer, 2006).
8. Qi, D, et al. Single-shot compressed ultrafast photography: a review. *Adv. Photon.* **2**, 014003 (2020).
9. Dabov K, Foi A, Katkovnik V, Egiazarian K. BM3D Image Denoising with Shape-Adaptive Principal Component Analysis. *SPARS - Signal Processing with Adaptive Sparse Structured Representations. Saint Malo, France.* (2009).
10. Boyd S, Parikh N, Chu E, Peleato B, Eckstein J. Distributed optimization and statistical learning via the alternating direction method of multipliers. *Found. Trends Mach. Learn.* **3**, 1-122 (2011).
11. Burger H, Schuler C, Harmeling S. Image denoising: can plain neural networks compete with BM3D? *CVPR*. 2392-2399 (2012).
12. Ramp function. https://en.wikipedia.org/wiki/Ramp_function
13. Chan S. Performance analysis of plug-and-play ADMM: a graph signal processing perspective. *IEEE Trans Comput Imaging* **5**, 274-286 (2019).
14. May P, Berry M. Tutorial on the acquisition, analysis, and interpretation of upconversion luminescence data. *Methods Appl. Fluoresc.* **7**, 023001 (2019).
15. Sillen, A, and Yves E. The correct use of “average” fluorescence parameters. *Photochem. Photobiol.* **67**, 475-486 (1998).
16. Boyer J, Vetrone F, Cuccia L, Capobianco J. Synthesis of colloidal upconverting $NaYF_4$ nanocrystals doped with Er^{3+}, Yb^{3+} and Tm^{3+}, Yb^{3+} via thermal decomposition of lanthanide trifluoroacetate precursors. *J. Am. Chem. Soc.* **128**, 7444-7445 (2006).
17. Brites C, Millán A, Carlos L. Lanthanides in luminescent thermometry. Handbook on the Physics and Chemistry of Rare Earths. Elsevier (2016).
18. Arakaki L.S., Burns D.H., and Kushmerick M.J., Accurate myoglobin oxygen saturation by optical spectroscopy measured in blood-perfused rat muscle. *Appl. Spectrosc.* **61**, 978-985 (2007).
19. Lin L., et al. In vivo photoacoustic tomography of myoglobin oxygen saturation. *J. Biomed. Opt.* **21**, 061002 (2015).
20. Liu L., et al. Fluorescence lifetime imaging microscopy using a streak camera. *SPIE Proc.* **8948**, 89482L (2014).

21. Gao L., et al., Single-shot compressed ultrafast photography at one hundred billion frames per second. *Nature*. **516**, 74-77(2014).
22. Liang J, et al. Single-shot real-time video recording of a photonic Mach cone induced by a scattered light pulse. *Sci. Adv.* **3**, e1601814 (2017).
23. Ma Y., et al., High-speed compressed-sensing fluorescence lifetime imaging microscopy of live cells. *Proc. Natl. Acad. Sci. U.S.A* **118**, e2004176118 (2021).
24. Wang P., Liang J., and Wang L., Single-shot ultrafast imaging attaining 70 trillion frames per second. *Nat. Commun.* **11**, 2091 (2020).
25. Wang C., et al., Line scanning mechanical streak camera for phosphorescence lifetime imaging. *Opt. Express*. **28**, 26717-26723 (2020).
26. Liu X, *et al.* Fast fluorescence lifetime imaging techniques: A review on challenge and development. *J. Innov. Opt. Health Sci.* **12** 1930003 (2019).

Supplementary Figures

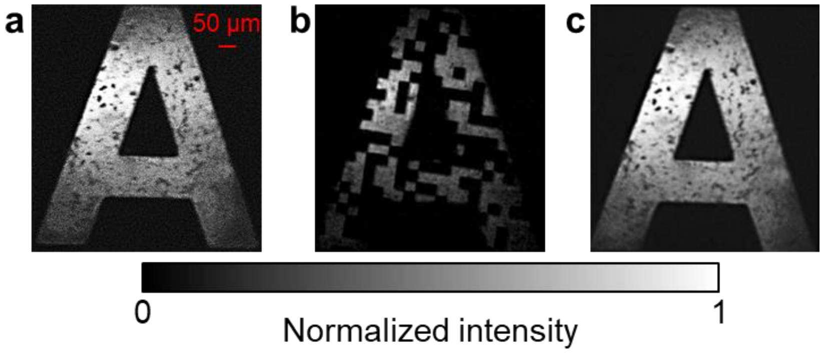

Supplementary Fig. 1. Image registration in SPLIT's dual-view data acquisition. a Image acquired in View 1. **b** Image acquired in View 2 without using optical shearing. **c** Co-registered image of View 1.

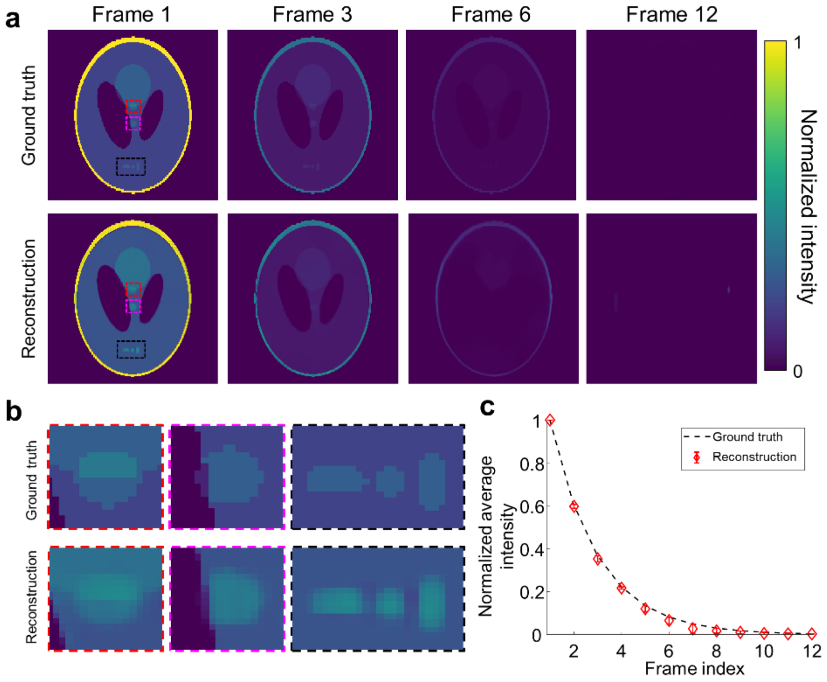

Supplementary Fig. 2. Simulation of the dual-view PnP-ADMM reconstruction algorithm. a Comparison of representative frames of the reconstructed result with the ground truth. **b** Comparison of three local features in Frame 1 of the reconstructed result with the ground truth (marked by the red, magenta, and black dashed boxes). **c** Normalized average intensity of the reconstructed result versus the frame index. Error bar: standard deviation.

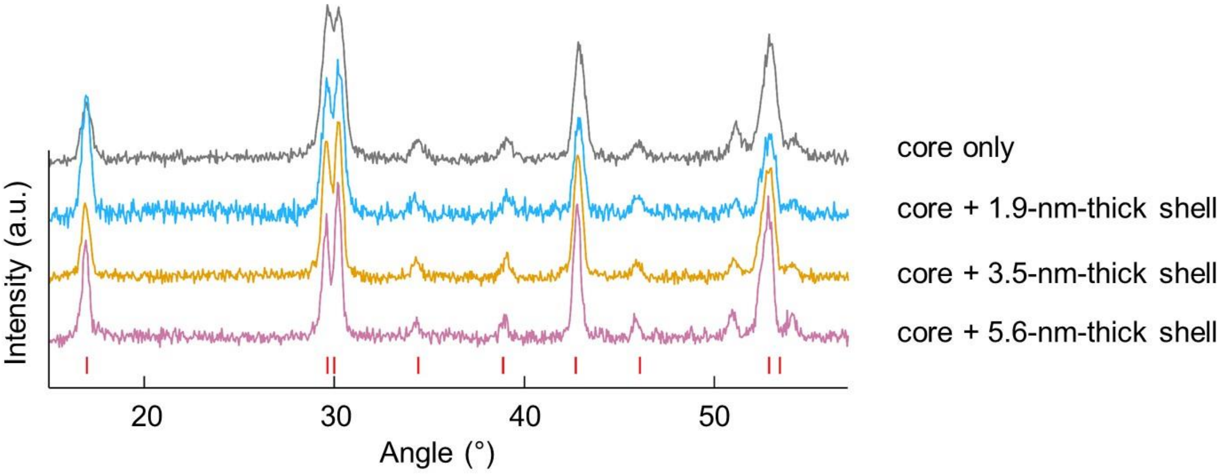

Supplementary Fig. 3. X-ray powder diffraction patterns of UCNPs. The core-only and core/shell $NaGdF_4:Er^{3+}$, $Yb^{3+}/NaGdF_4$ UCNPs following their growth by increasing the shell thickness. Red lines: Diffraction peaks of pure hexagonal $NaGdF_4$ (data are taken from PDF# 01-080-8787).

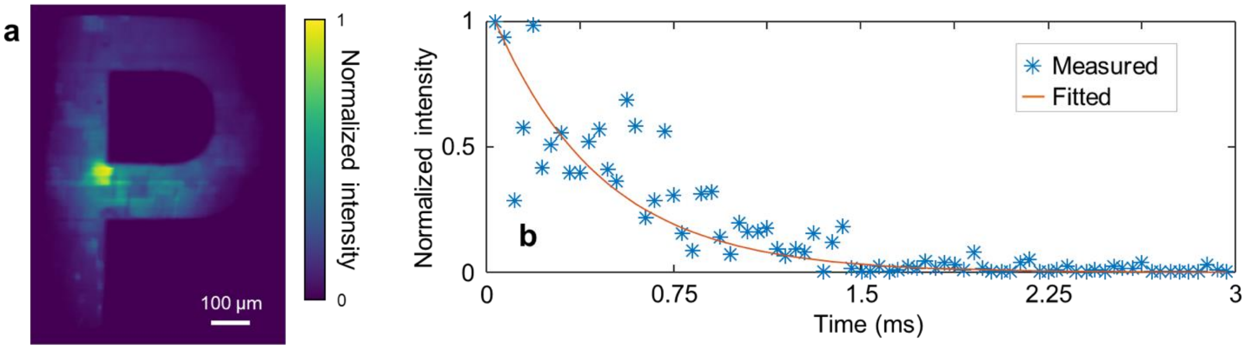

Supplementary Fig. 4. Characterization sensitivity of the SPLIT system. a Temporally integrated reconstructed image at the excitation laser power density of 0.06 W mm^{-2}. **b** Normalized intensity as a function of time with a fitting curve.

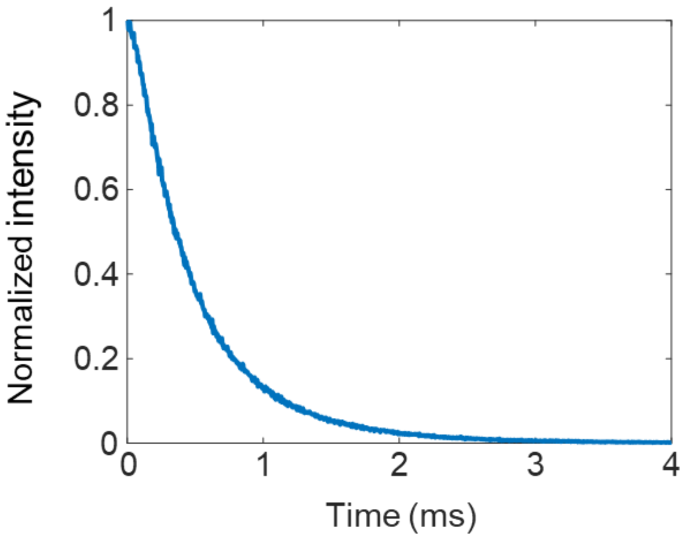

Supplementary Fig. 5. Measurement of the green upconversion emission lifetime of the 5.6 nm-thick-shell UCNPs using the TCSPC method.

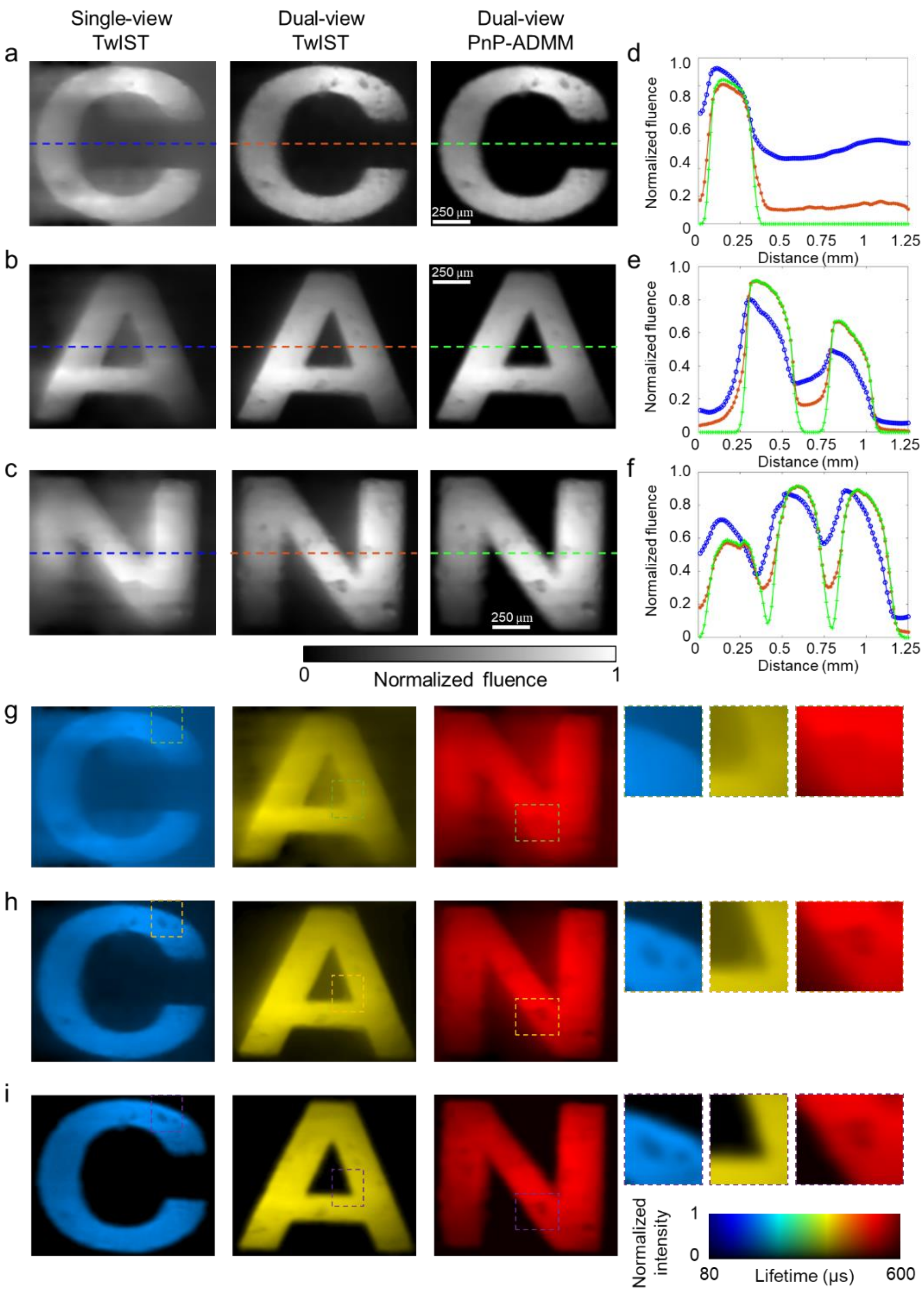

Supplementary Fig. 6. Comparison of quality of images reconstructed by using different algorithms. a Letter “C” reconstructed by using the single-view TwIST, dual-view TwIST, and dual-view PnP-ADMM algorithms, respectively. **b-c** As (a), but for letters “A” and “N”. **d** Comparison of the selected line profiles of the reconstructed images of letter “C”. **e-f** As (d), but for letters “A” and “N”. **g-i** Lifetime maps of the three letters produced by the single-view TwIST (g), single-view PnP-ADMM (h), and dual-view PnP-ADMM (i) algorithms. Insets: zoom-in views of three local areas.

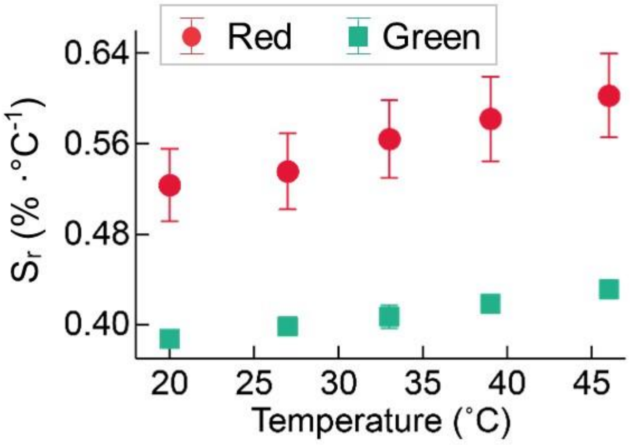

Supplementary Fig. 7. Quantification of relative temperature sensitivities of the green and red emissions of the core/shell $NaGdF_4$:Er^{3+},Yb^{3+}/$NaGdF_4$ UCNPs with a 5.6 nm-thick shell. Error bar: standard deviation.

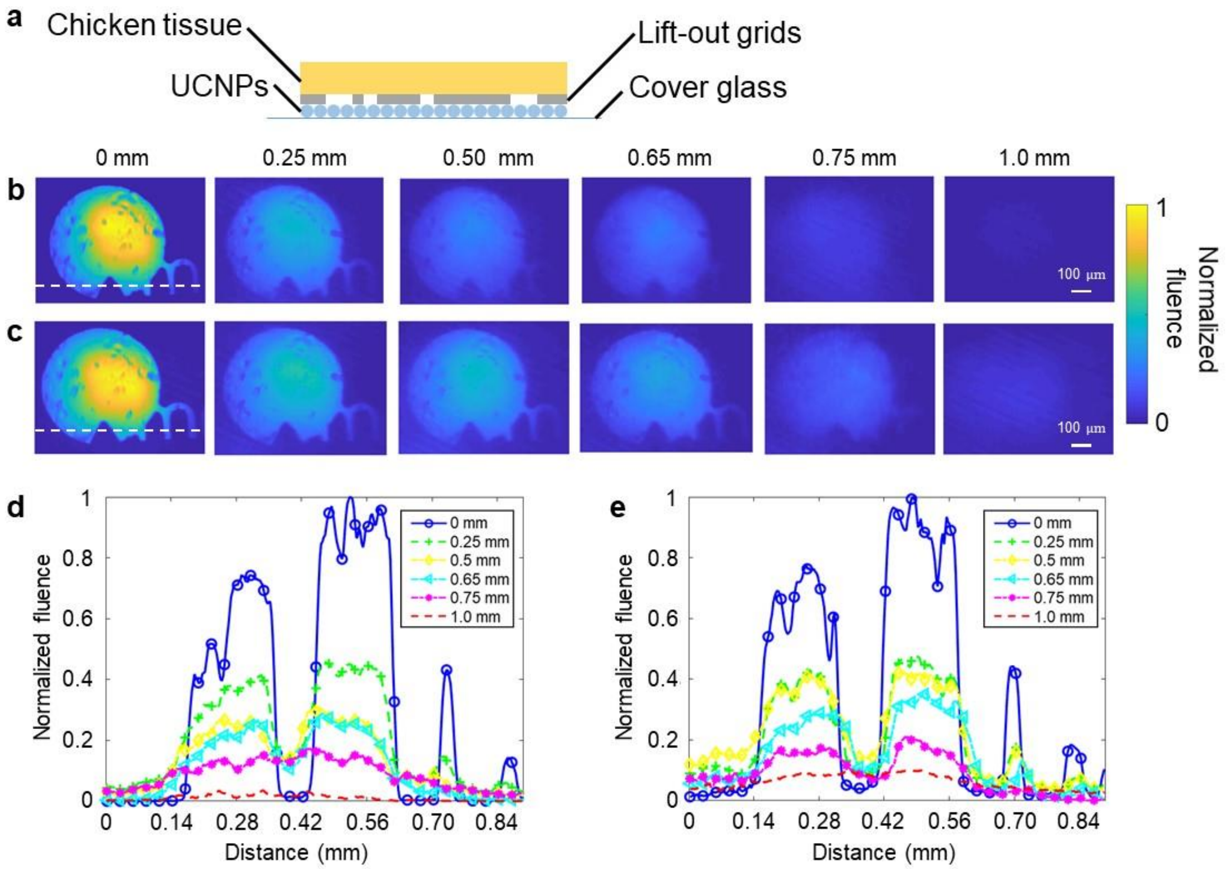

Supplementary Fig. 8. Demonstration of SPLIT with a fresh chicken tissue phantom. a Experimental setup. **b** Temporally projected images of the reconstructed dynamic scene at the depths from 0 to 1 mm with the green emission. **c** As (b), but for the red emission. **d** Comparison of normalized fluence of a representative cross-section [marked by the white dashed line in the first panel in (b)] for various imaging depths. **e** As (d), but for the red emission. The representative cross-section is marked by the white dashed line in the first panel in (c).

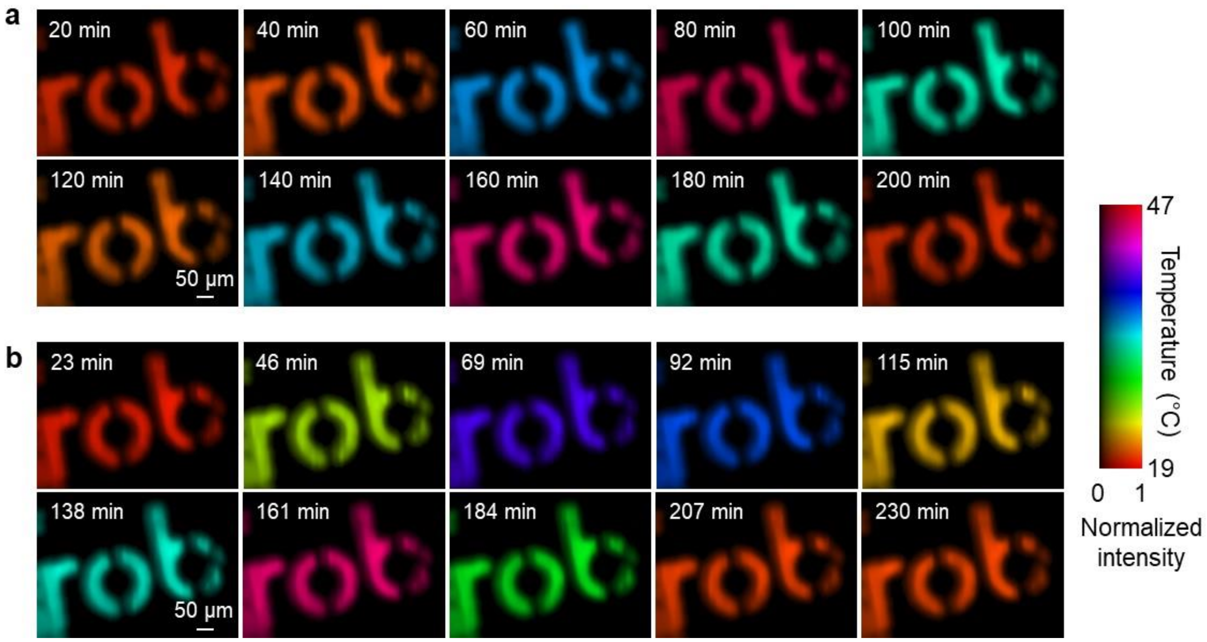

Supplementary Fig. 9. Longitudinal temperature monitoring using green (a) and red (b) luminescence emissions from the 5.6 nm-thick UCNPs covered by a transmissive mask of letters "rob".

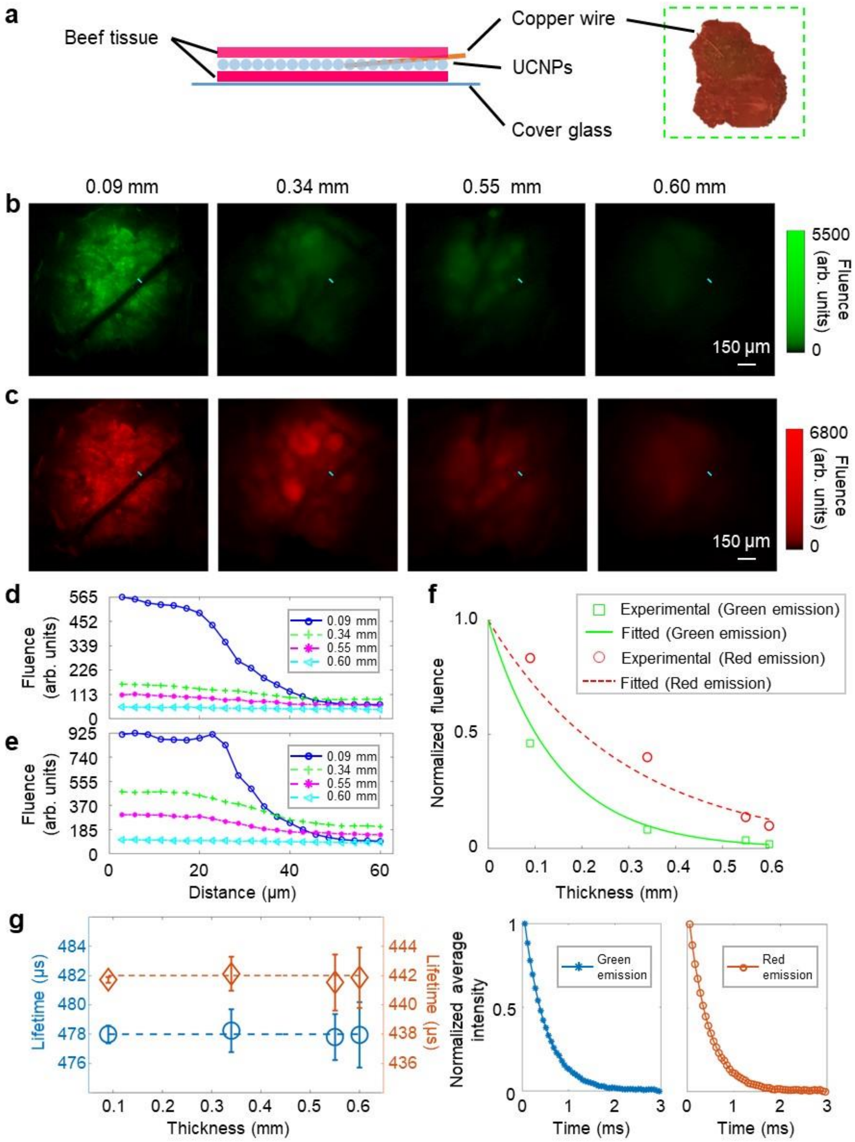

Supplementary Fig. 10. Demonstration of SPLIT with a fresh beef tissue phantom. a Sample preparation. **b** Temporally projected images of the reconstructed dynamic scenes at the depths from 0.09 to 0.60 mm for the green emission. **c** As (b), but for the red emission. **d–e** Cross-sections of a selected spatial feature [marked by the light blue solid line in (b) and (c)] for various depths for the green emission (d) and the red emission (e). **f** Normalized fluence versus tissue thickness for the green and red emissions with single-component exponential fitting. **g** Lifetimes as the function of the thickness for the green emission (blue circles; the mean value is plotted as the blue dashed line) and the red emission (orange diamonds; the mean value is plotted as the orange dashed line). Error bar: standard deviation. Right insets show the decay of normalized average intensity at the depth of 0.09 mm for the green and red emissions, respectively.

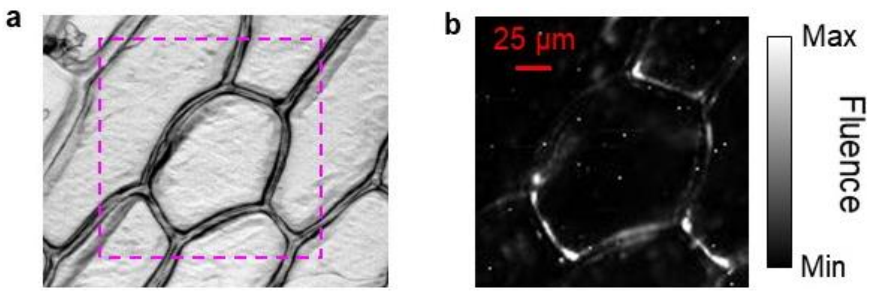

Supplementary Fig. 11. Single-layer onion cell sample. a Image of the sample taken by a bright-field microscope. **b** Confocal microscopy of green upconversion emission of UCNPs diffused in an individual onion cell [marked by the magenta dashed box in (a)].

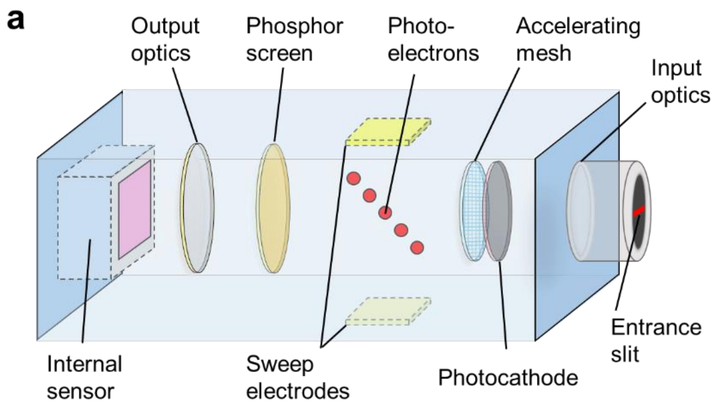

Supplementary Fig. 12. Schematics of an optoelectronic streak camera (a) and a mechanical streak camera (b) in their conventional operations.

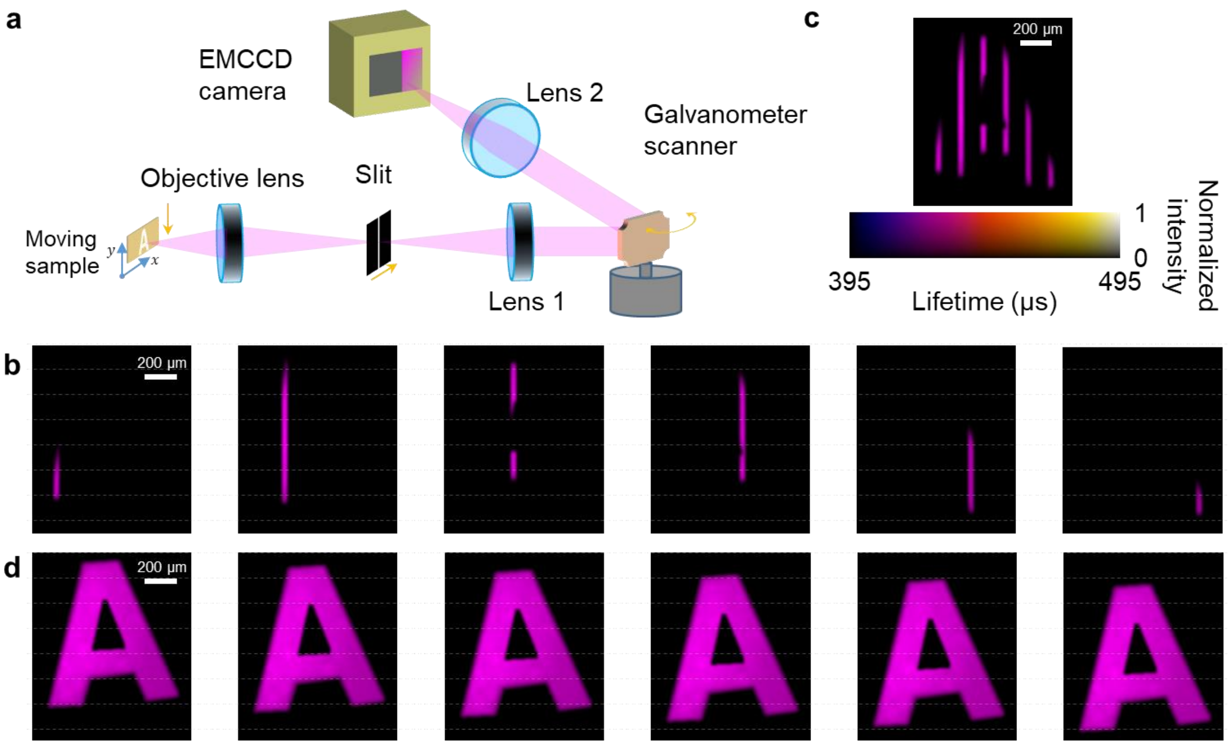

Supplementary Fig. 13. Comparison between line-scanning microscopy and SPLIT in 2D PLI capability. a Experimental setup of line-scanning microscopy. The moving UCNPs sample was loaded onto a translation stage. The moving directions of the sample and the slit are marked by orange arrows. **b** 1D photoluminescence lifetime images produced by using the line-scanning setup. **c** Distorted partial 2D lifetime map synthesized by using the data in (b). **d** Six 2D lifetime maps of the sample moving downward captured by using the SPLIT system.

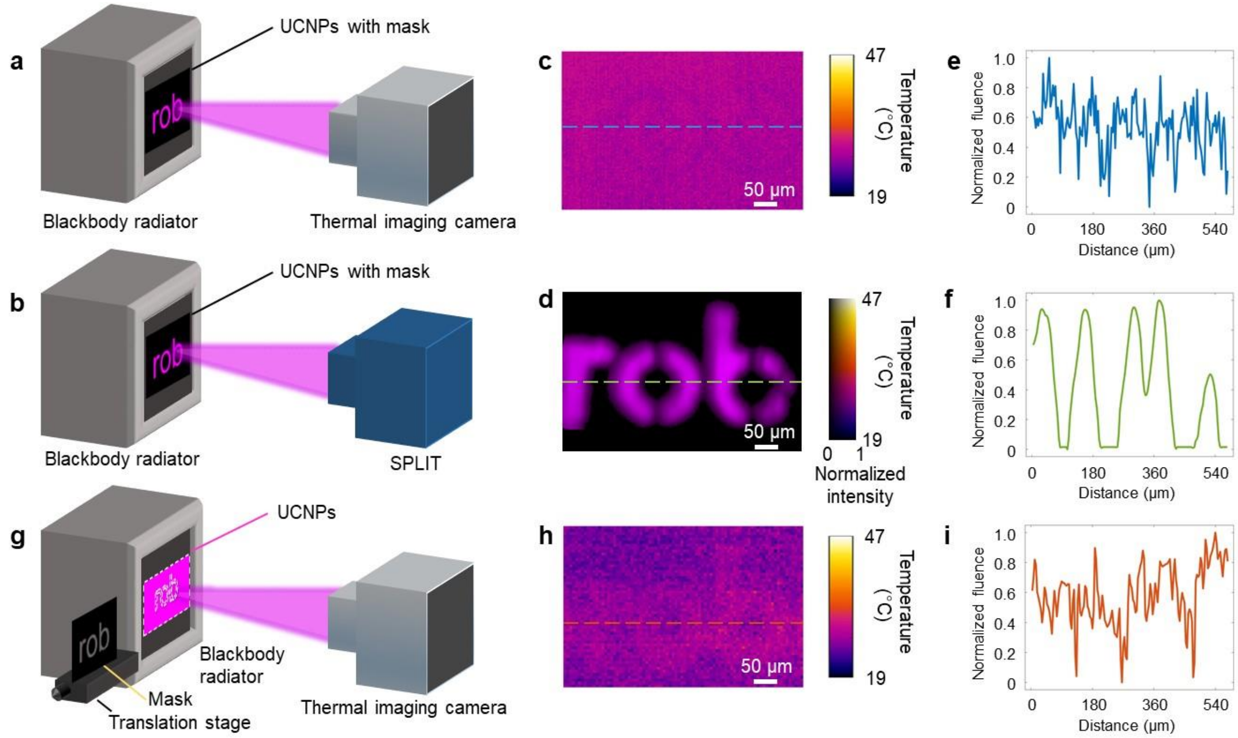

Supplementary Fig. 14. Comparison between the thermal imaging camera and SPLIT in temperature imaging. a–b Experimental setup using thermal imaging camera (a) or SPLIT (b). The sample and mask were heated up by a blackbody radiator. **c** Temperature image captured by using the thermal imaging camera. **d** As (c), but using SPLIT. **e–f** Selected line profile from (c) and (d), respectively. **g** As (a), but using a translation stage to move the mask with the room temperature. **h** Temperature image captured by the setup in (g). **i** Selected line profile from (h).

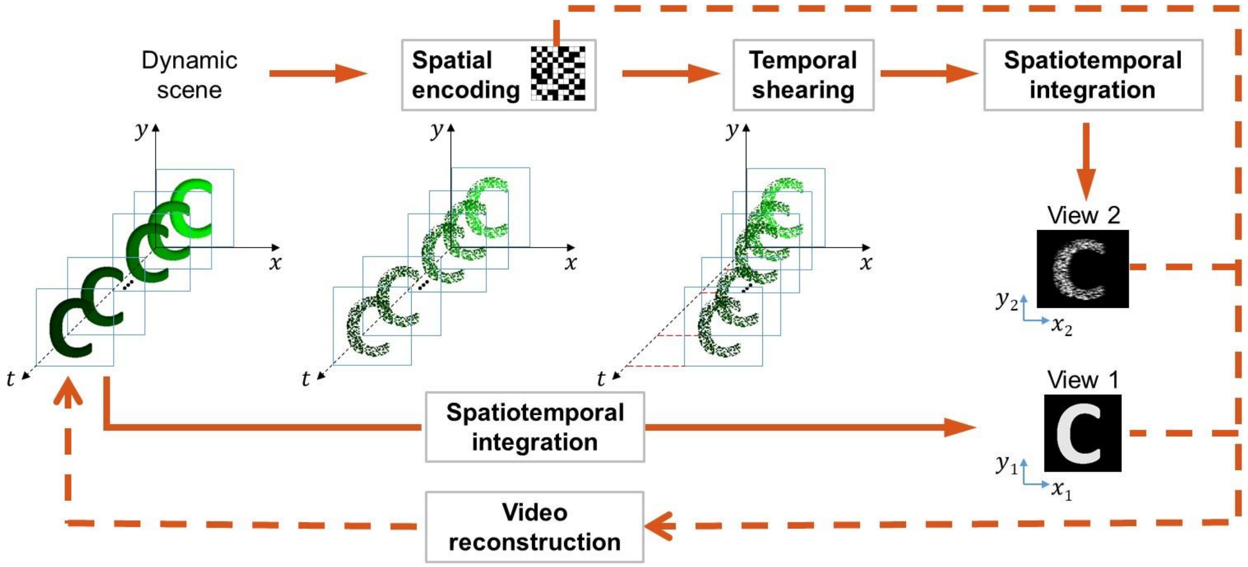

Supplementary Fig. 15. Illustration of the working principle of SPLIT.

Supplementary Table

Supplementary Table 1. Comparison of representative 2D lifetime imaging modalities using streak cameras

	CUP [21]	LLE-CUP [22]	CUSP [24]	Sequential 1D ultrafast photography [20]	Sequential 1D ultrahigh-speed photography [25]	SPLIT
Application	Macroscopic FLI	High-speed FLIM [23]	Spectrally resolved FLIM	FLIM [26]	PpLIM	Video-rate PLI thermometry
Imaging scheme	Compressed streak imaging	Compressed streak imaging	Compressed streak imaging	Line-scanning	Line-scanning	Compressed streak imaging
Single-shot 2D mapping	Yes	Yes	Yes	No	No	Yes
Throughput	High	High	High	Low	Low	High
Inter-frame interval	20 ps	40 – 500 ps	2 ps	~6 ps	5.8 ns – 4.7 μs	30 – 250 μs
Recording time window	~5 ns	20 – 100 ns	0.8 ns	6 ns	52 ns – ~1 ms	3 ms
Spatial resolution	1.7 mm	≥ 0.2 μm	50 μm	~0.7 μm	~0.4 μm	20 μm
Applicability to dynamic samples	Yes	Yes	Yes	No	No	Yes
Type of streak camera	Optoelectronic	Optoelectronic	Optoelectronic	Optoelectronic	Mechanical	Mechanical
Cost	High	High	High	High	Low	Low
Size	Big	Big	Big	Big	Small	Small
Quantum efficiency (QE)	Moderate	Moderate	Moderate	Moderate	High	High
Power consumption	High	High	High	High	Low	Low

Note: CUP, compressed ultrafast photography; CUSP, compressed ultrafast spectral photography; FLIM, fluorescence lifetime imaging; LLE, lossless-encoding; PpLIM, phosphorescence lifetime imaging microscopy.
