# Supplementary material for: Fast wide-field upconversion luminescence lifetime thermometry enabled by single-shot compressed ultrahigh-speed imaging
Source: Nat Commun. 2021 Nov 4;12:6401. doi: 10.1038/s41467-021-26701-1 (PMC8568918; doi:10.1038/s41467-021-26701-1)
Supplement: Supplementary file 3 — Description of Additional Supplementary Files [file 41467_2021_26701_MOESM3_ESM.docx]

**Description of Additional Supplementary Files**

**File Name:** Supplementary Movie 1

**Description:** Characterization of the SPLIT system’s spatial resolution by imaging photoluminescence intensity decay of UCNPs covered by a negative resolution target.

**File Name:** Supplementary Movie 2

**Description:** Wide-field photoluminescence intensity decay of three types of core/shell UCNPs covered with transparencies of letters “C”, “A”, and “N”.

**File Name:** Supplementary Movie 3

**Description:** Intensity decay of green (top row) and red (bottom row) luminescence emissions of 5.6 nm-thick-shell UCNPs covered with transmissive patterns at five different temperatures.

**File Name:** Supplementary Movie 4

**Description:** Intensity decay of green and red emissions of a transmissive pattern beneath fresh chicken tissue with different thicknesses.

**File Name:** Supplementary Movie 5

**Description:** Longitudinal temperature monitoring using the green and red emissions. The sample was 5.6 nm-thick-shell UCNPs covered with transmissive pattern “rob” beneath 0.5 mm-thick fresh chicken tissue.

**File Name:** Supplementary Movie 6

**Description:** Video-rate temperature mapping of moving single-layer onion cells.
